# Threshold Differences on Figure and Ground: Gelb and Granit (1923)

**DOI:** 10.1177/2041669516685722

**Published:** 2017-01-01

**Authors:** Max Kinateder, Rolf Nelson

**Affiliations:** Department of Psychological and Brain Sciences, Dartmouth College, Hanover, NH, USA; Department of Psychology, Wheaton College, Norton, MA, USA

**Keywords:** attention, contours or surfaces, perceptual organization, shapes or objects

## Abstract

In 1923, Gelb and Granit, using a method of adjustment for a small red light, reported a lower threshold for the target when presented on a ground region than on an adjacent figural region. More recent work in perceptual organization has found precisely the opposite—a processing advantage seems to go to items presented on the figure, not the ground. Although Gelb and Granit continue to be cited for their finding, it has not previously been available as an English translation. Understanding their methodology and results is important for integrating early Gestalt theory with more recent investigations.

## Introduction

### Adhémar Gelb and Ragnar Granit

Both Adhémer Gelb (1887–1936) and Ragnar Granit (1900–1991) had long and distinguished careers in research. Gelb, the senior of the two, was born in Russia but spent the majority of his research career at the University of Frankfurt. He was well known for his collaborative nature, and he was highly influential in the circle of Gestalt perceptual psychologists in Germany at the time. He is now perhaps best known for the eponymous Gelb Effect, a demonstration of the effect of the surround on brightness perception. He continued his work at Frankfurt until 1933, when he was dismissed by the Nazis and, after a period of exile, died under poor conditions in a German sanatorium (Devonis, 2012).

Granit, only 23 when his collaboration with Gelb was published, was born in Finland and was in the process of completing his medical studies in Helsinki with a focus on visual psychophysics. In the following years, he would go on to have an extremely long and productive career. His most celebrated work was in two areas: the physiology of the motor system, for which he was nominated for a Nobel prize, and for the mechanisms of color vision, for which he won the Nobel prize in 1967. In addition to his substantial and influential corpus, he was a dedicated teacher and mentor, training a generation of Swedish scientists while heading the Nobel Institute for Neurophysiology (Kernell, 2000).

### Their Finding

The basic finding of Gelb and Granit’s (1923) article is fairly straightforward: A small projected red dot was more easily detected on the ground than on the figure. The stimulus they used is shown in their Figure 1: A fan-shaped figure against a surrounding homogenous ground. Using an ingenious setup (Figure 2), the observer was allowed to control the brightness of the red target by moving the illuminant nearer or farther along a slider. All necessary control conditions in terms of brightness of the figure and ground—contrast with the target—were done by including negative (inverse) images. Thresholds were determined by a method of adjustment, though ascending only.

**Figure 1.**
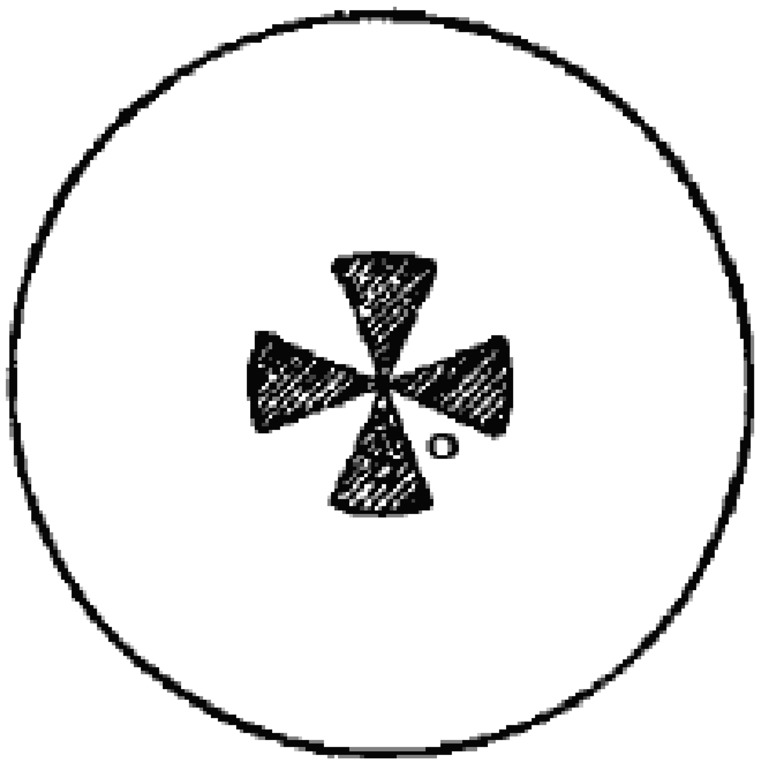
Figure-ground stimulus used in the experiment.

**Figure 2.**
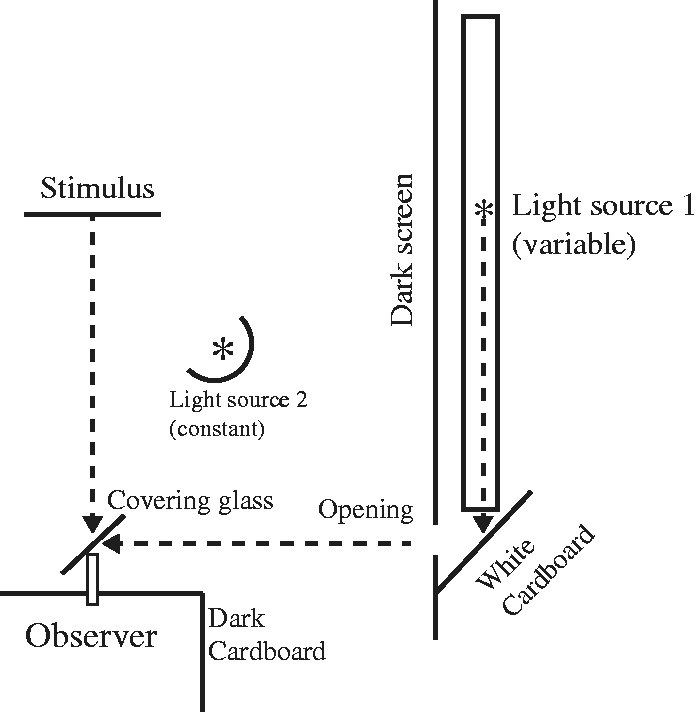
The essential details of the experimental setup are illustrated.

Their explanation of these results was based on theoretical accounts by Rubin (1920) and Köhler (1920). Köhler, as cited in their article, considered the figure to be more vivid than the ground, and that the figure had a “higher psychophysical energy density”; Gelb and Granit suggested that the figure was a “more vivid psychophysical event.” They claimed that when the target (red dot) appeared, it became the new figure. Because there should be more *resistance* from a region that was previously figure than from a region that was already ground, it should be harder to detect on the (previous) figure.

Interestingly, they also considered the role that attention might have played in their experiment. In fact, they took it as obvious that a figure attracts more attention than a ground. In their interpretation, their results are all the more evidence that the figure is more tightly bound than a ground because attentional effects should have attracted attention to the figure. However, it should be noted that because their stimuli appeared for an extended time period, and observers knew in advance where the target would appear, that attention would likely have played a limited role.

### Subsequent Research

Current research on figure–ground organization is less focused on the phenomenological status of figures versus grounds and has rather been understood as the process of border ownership at a contour. The side that owns the contour is the figure in the sense that it is perceived to be in front and to be shaped, providing input for later perceptual processes.

Recent studies have suggested, in contrast to Gelb and Granit, that there is a processing advantage for the *figural* region. Wong and Weisstein (1982) demonstrated superior discrimination for targets appearing in the perceived figure in a bistable face or vase stimulus. Nelson and Palmer (2007) showed an advantage for both detectability and discrimination in the figural region (defined by meaningfulness of the contour) after a sudden onset. These effects were interpreted to mean that attention was automatically drawn to the figural region, consistent with accounts of automatic attentional allocation to the onset of a new perceptual object (Yantis & Jonides, 1984). Salvagio, Cacciamani, and Peterson (2012) found poorer orientation discrimination on the ground than on the figure, which they interpreted as a ground suppression effect. In another series of experiments (Hecht, Cosman, & Vecera, 2016; Lester, Hecht, & Vecera, 2009), several types of perceptual enhancements were found for figural regions, including an earlier perceived onset and a later perceived offset, while ground regions were suppressed.

Neural investigations into figure or ground organization have been congruent with these recent behavioral findings. Single cells in early visual cortex (V2) can code for border ownership (Zhou, Friedman, & von der Heydt, 2000), and recent evidence has shown figural enhancement and ground suppression in V1 and V4 cells (Poort, Self, van Vugt, Malkki, & Roelfsema, 2016). In addition, electrophysiological studies have indicated that figural regions evoke stronger steady-state potentials (Brooks & Palmer, 2011) and are preferentially routed to object recognition areas in lateral occipital areas (Applebaum, Wade, Vildavski, Pettet, & Norcia, 2006).

It is currently unclear what is responsible for the discrepancy between Gelb and Granit and more recent findings, but it may be that perceptual or attentional effects in figure–ground displays are highly sensitive to particular experimental conditions. Among these might be the timeframe (Gelb & Granit left displays up for extended viewing), awareness of experimental conditions, effects of nearby contours (e.g., in Gelb & Granit’s displays, the target was proximate to more contours on the figural side), and the nature of the target. If results are sensitive to these and other factors, it is important for current researchers to investigate them systematically.

Gelb and Granit’s work on threshold differences between figure and ground did much to frame early theory on perceptual organization. Koffka, in his influential *Principles of Gestalt Psychology* (1935), declared that Gelb and Granit had provided definitive evidence that figures are more strongly organized than grounds. With this translation, we hope to usefully integrate an important finding from early Gestalt research with modern experimental evidence on the consequences of figure–ground assignment. Because processing differences between figure and ground continue to be a subject of debate, Gelb and Granit’s work remains relevant almost 100 years after its publication.

## The Role of “Figure” and “Ground” for Color Thresholds

### Abridged translation of Gelb & Granit (1923).^1^ Full translation provided as Supplementary Materials.

Imagine the following threshold experiment: In one case, the gray patch (to which the color is to be added) is presented as a homogenous plane covering the entire field of view; in the other case, the patch is presented with the same (objective and subjective) brightness, but as a ring shape on a rotating disk.^2^ Apart from questions regarding brightness and expanse, there is a fundamental difference between the two patches. The ring shape appears as a self-contained unit that delimits itself from the inside and the outside: The ring stands out as a *figure* from a brighter or darker *ground*.^3^ In turn, the homogenous patch, which covers the entire field of view, appears evenly and uniform in general and does not, or at least in the same sense, appear as a *figure*; it is missing a brightness difference, without which seeing a figure in its actual sense is not possible.

These examples may seem extreme. However, the characteristic differences remain, although not always as pronounced, under similar conditions: A gray patch formed by an entire rotating disk is—again independent from brightness and expanse—phenomenally different than one that has the shape of a ring on a rotating disk. In the latter case, the surrounding of the ring (figure) has a distinctive ground character; in the former, it does not. Likewise, a ring that does not differ strongly from the background is phenomenally a different patch than a ring with the same brightness that stands out from the background: In the latter case, the gray patch is a *much better figure* and its background has a *clear ground character*. Generally speaking, any gray patch selected for threshold studies can be described as either having more figure or more ground features.

Results reported by Rubin (1920) and elaborations by Köhler (1920) highlight the fundamental importance of figure–ground differentiation. This difference is not only phenomenological in nature, but, as Rubin showed in a series of experiments, two objectively identical patches exert fundamentally different psychophysical effects depending on whether they are experienced as figure or as ground.

Since the gray patch presented in studies on color thresholds has either more or less ground character, the question arises as to whether the color thresholds themselves vary depending on whether the gray patch has more features of ground or of figure. If dependencies between the just noticeable color impression and figure–ground experiences exist, these should have a significant impact on color theory. The specific question of the present study is: Is the color threshold (in a gray patch of a certain objective brightness) for a given intensity of a color stimulus different if the patch appears either as *figure* or as *ground*?

### Methods and Procedure

We used photographic techniques to produce the gray patches to which we added the color stimuli.^4^ We used images showing a figure resembling a Maltese cross of about 5 cm diameter on a ground of about 15 cm diameter (see Figure 1). The size of each of the four wings of the cross and each of the four gaps corresponded to an eighth of a circle. The cross—the figure—was either darker or lighter than the ground. We will refer to images with a darker figure as *positive images* and to images with a lighter figure as *negative images*.

We used four different positive images, which varied in the brightness of figure and ground as well as in the brightness between figure and ground. Each of the four positive images had a corresponding negative image: For example, if the brightness of the figure was a mix of 340° black + 20° white produced on a disk, and the brightness of the ground was 36° black + 324° white, the figure in the corresponding negative images was 36° black + 324° white, and the ground was 340° black + 20° white.

We derived an equation between each patch and a reference disk consisting of black (Tuchschwarz^5^) and white (barium white) sectors, to identify the brightness of the different patches. The reference disk and the patches were placed next to each other were observed from behind a screen with a hole at an appropriate distance, allowing observers to see only a small fraction of the reference disk and the patch. Brightness was considered as equal if the hole in the screen appeared to be of a completely homogenous, color neutral quality.

The following table provides a description of the different levels of brightness used in the study. Constellations I to IV refer to the four different positive images and corresponding negative images and describe the relation of figure to ground brightness for both positive and negative images. For the sake of brevity, only the black proportions are shown.
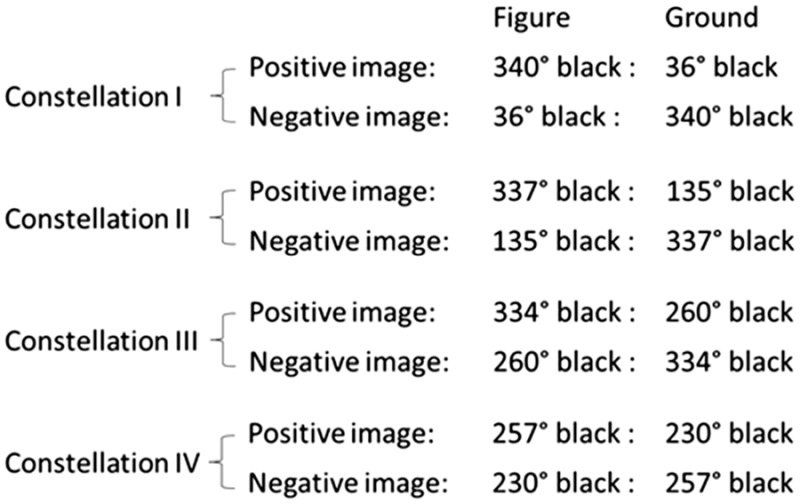


There were the following descriptive differences between these four constellations: In Constellation I, the difference between figure and ground appeared phenomenally the strongest, in Constellation IV the weakest. Constellations II and III were in the middle; however, Constellation II was more similar to Constellation I, and Constellation III was more similar to Constellation IV.

To investigate how the color threshold changes when the color stimulus is mixed with either a figure or a ground of a certain brightness, thresholds were determined for the figure (ground) of a positive image and then for the ground (figure) of the corresponding negative image. The values attained were then compared with each other.

The color thresholds were determined for a defined area of 3 mm diameter located as shown in Figure 1 on figure and ground (the two small circles). The experiment used the following procedure.

The observer looked at the photograph which was placed in about 1 m distance, frontoparallel, and at eye-level. He looked through a monocular tube which only allowed viewing figure and ground, where the ground appeared to expand in all directions. A fairly large covering glass was placed in front of the tube opening facing the image; its (the glass’s) location was set so that a fraction of colored light coming from an adjustable light source at the side was mirrored into the observer’s eye. That is, the color stimulus was mixed to the figure or ground by adding the colored light to the colorless light emitted by the image.

An electric lamp of 16 Normalkerzen^6^ (Light Source 1) was placed on a slider and could be moved easily and without noise.^7^ A fraction of the light was reflected on a dark screen with a 3 mm opening by a white piece of cardboard which was placed in a constant position at an angle of the lamp’s moving trajectory. A gelatine light filter was placed in front of the opening on the side facing the covering glass. The photograph was illuminated by a constant light source (Light Source 2) invisible to the observer and was of course placed at the same distance from the covering glass as the opening.

The intensity of the colored light was gradually decreased by changing the distance of the lamp to the reflecting cardboard; the distance of the lamp to the cardboard could be measured on a scale from 0 to 100 cm with a precision of 1 mm. Although this procedure was not sufficiently exact for our lamp (which radiated in all directions), we still used this method since we were only interested in the relative comparison between figure and ground thresholds and not in the numerical values (of the thresholds).

The experiments were conducted in a semidarkened room. When the experimenter called *now*, the observer, whose head was fixated by a chin-rest, was to look at the location of color stimulus (the center of the lower crosswing or the center of the right lower gap). The observer moved the light on the slider and then signaled to the experimenter the moment a true color impression became just visible. Participants were specifically instructed to only signal when they saw a color impression and not if a colorless or an unspecified spot became visible.

For each threshold measurement to be under the same adaptation conditions, the observer had to look into a semidark corner made out of two pieces of dark cardboard immediately after the end of each trial (see Figure 2).

To be able to safely compare the figure thresholds to the ground thresholds, color thresholds for each of the four constellations were identified in a single session. To avoid potential influence of practice or fatigue on the results, we completed the threshold measurements within each constellation in the following order: If the color stimulus was mixed to the dark patch, the threshold was first measured for the figure then for the ground; if the color stimulus was mixed to the light patch, the thresholds were measured in inverse order.

We only used the ascending method: Beginning at a position of the lamp at which no color was perceived, the lamp was gradually moved closer to the reflecting cardboard and the position was identified at which the color was first recognized (the sliding or moving of the lamp followed a mean speed which varied from case to case). Initially we also used the descending method, however, we decided to discard this method since it was subjectively felt to be unreliable and objectively lead to larger variance in the data.

As we were—at least initially—not interested in studying color thresholds for different colors, we almost exclusively used red light. In addition, we ran experiments using green light, but more for control purposes and only using Constellation I. We used gelatine light filter#25 (“scarlet”) and #14 (“methyl green and picric acid”) provided by Dr. Steeg and Reuter in Homburg v.d.H. The filtered light, especially by the red filter, was tested with an objective spectrum and found to be very pure.

In addition to us (Gb. and Gr.) the following gentlemen participated in the study: Mr. stud. phil. Wenzel (Wz.), Mr. stud. phil. Schriever (Schr.), Mr. stud. phil. Steuerwald (St.), Mr. stud. phil. Greb (Grb.), and Mr. Wingenbach (Wgb.). None of the gentlemen, whom we would like to thank for their participation, were familiar with the research question.

### Results

Table I shows the threshold values for red, consisting of mean values from five single trials. Prior to these trials, participants completed three to five trials (not reported in the table) in which participants practiced the task (and were acclimated to the procedure).

**Table 1.** Results.

		Constellation I 340° Black: 36° Black						Constellation II 337° Black: 135° Black						Constellation III 334° Black: 260° Black						Constellation IV 257° Black: 230° Black					
Observer		DF	DG	Q_D_	LF	LG	Q_L_	DF	DG	Q_D_	LF	LG	Q_L_	DF	DG	Q_D_	LF	LG	Q_L_	DF	DG	Q_D_	LF	LG	Q_L_
Gb.	cm	63.4	77.3		30.7	32.7		59.9	71.4		32.3	34.3		61.5	70.4		47.1	50.9		47.2	51.2		34.7	36.1	
	mV	0.3	1.12		0.68	0.48		0.66	2.06		1.1	0.92		0.5	0.83		0.66	1.16		0.32	0.12		0.18	0.3	
	LE	2.48	1.68	1.48	10.57	9.35	1.13	2.78	1.96	1.41	9.58	8.51	1.12	2.64	2.0	1.32	4.51	3.85	1.17	4.48	3.82	1.17	8.30	7.67	1.08
Gr.	cm	66.2	89.3		36.6	38.7		53.4	89.6		40.8	43.0		63.3	82.2		52.3	69.8		55.8	58.5		54.0	59.6	
	mV	0.48	0.1		0.2	0.24		0.48	0.16		0.26	0.2		0.24	0.24		0.24	0.18		0.32	0.3		0.23	0.16	
	LE	2.28	1.15	2	7.45	4.49	1.11	3.49	1.23	2.83	6.0	5.38	1.11	2.49	1.48	1.48	3.61	2.05	1.76	3.24	2.90	1.1	3.4	2.8	1.2
Wz.	cm	52.7	91.8		30.7	33.9		64.4	95.4		41.5	46.0		72.3	97.5		58.9	62.1		72.6	95.7		69.4	72.9	
	mV	0.46	0.72		0.24	0.7		0.28	0.46		0.22	0.08		0.58	0.6		0.4	1.28		0.44	0.52		0.54	0.34	
	LE	3.6	1.2	3	10.6	8.7	1.22	2.42	1.09	2.22	5.81	4.7	1.23	1.9	1.05	1.8	2.87	2.59	1.1	1.89	1.09	1.83	2.12	1.88	1.13
Schr.	cm	61.2	73.8		29.5	31.5														50.8	59.8		41.2	43.5	
	mV	0.48	0.36		0.22	0.16														0.54	0.3		0.2	0.32	
	LE	2.67	1.84	1.45	11.5	10.1	1.14													3.87	2.8	1.38	5.9	5.2	1.13

The table should be read as follows:

Rows: The first row contains the distance of the lamp to the reflective cardboard in cm (compare with the sketch in Figure 2). The second row contains the corresponding mean variations (m.V.); in the third row we report the values of the first column in the corresponding light units (L.E.), by setting the light intensity of the cardboard at 1 when the lamp was a distance of 1 m from the reflecting cardboard.^8^ That is, the light intensity of the cardboard served as a reference point for the threshold values.

Columns: Columns labeled D.F. (“dark figure”) refer to thresholds for constellations with a darker figure and a lighter ground, i.e. figures with 340° black, 337° black, 334° black, and 257° black. D.G. (“dark ground”) refers to thresholds with a corresponding darker ground. The third row, labeled Q_D_ reports the proportion of the figure and ground threshold values.

Columns labeled L.F. (“light figure”) and L.G. (“light ground”) refer to threshold values for cases in which the lighter figure and ground, i.e. patches with 36° black, 135° black, 260° black, and 230° black; Q_L_ refers to the corresponding proportions.

The following conclusion can be drawn from Table I:

1. First, confirming our previous results, adding a color stimulus to a whiter patch led to higher color thresholds than adding a color stimulus to a blacker one. If one finds a deviation from this rule with participant Wz., one has to consider that the chosen experimental paradigm only allows for a comparison between thresholds within a constellation. In addition, practice and experience seem to have played a role with this participant.
2. Second, the main findings are as follows: The color threshold of any given patch of objectively same brightness varied depending on whether the respective field was seen either as figure or as ground. The figure threshold was higher than the ground threshold, independent from the objective brightness (all q_d_ and q_h_ values are bigger than 1).
3. Third, this observation was more pronounced for the darker patch compared with the lighter patch within each constellation: The q_d_ value of a constellation was, on average, larger than the corresponding q_h_ value. The only exception was the values in Constellations III and IV of participant Gr.
4. Fourth, a closer look at Table I reveals that the influence of figure–ground on the color threshold varied over different constellations.

Compare the q_d_ and q_L_ values for Constellations I to III. Despite the relatively small differences in objective brightness of Constellations I and III (340° black in Constellation I vs. 334° black in Constellation III), the q_L_ values, for instance, in Constellation I (participants Gb., Gr., Wz.) are noticeably smaller than in Constellation III. One could assume that the lower q_L_ values in Constellation III are the result of a brighter patch than in Constellation I. This assumption could be further supported by the notion that the subjective brightness of the patch in Constellation III is lower than in Constellation I as a result of adding less black to the color mix. However, this would hardly be in accordance with the observation that the participant Gb.’s q_L_ values in Constellation I (1.13) are only marginally smaller than the q_L_ values in Constellation III (1.17), although Constellation I (36° black) is objectively significantly brighter than Constellation III (260° black). In addition, the stronger contrast in brightness in Constellation I increases the subjective brightness of the field even more. Further, this would not be in accordance with the q_L_ value of 1.22 (participant Wz.) being even larger than as the q_L_ value of 1.1 in Constellation III (we have to ignore the the q_L_ value of 1.76 of participant Gr. in Constellation III at this point as he had been identified as an outlier earlier). Equally hard to understand would be why the q_L_ values in Constellation I (participant Gb., Gr., Wz.) almost completely match the q_L_ values in Constellation II, although the corresponding patches still differ considerably in objective brightness (36° black in Constellation I and 135° black in Constellation II). It would also remain unclear why the q_D_ values of participants Gb. and Wz. in Constellation II are smaller than in Constellation I, although the proportion of white is comparable in both cases (340° black and 337° black), and why the q_D_ values of 2.83 (participant Gr.) in Constellation II are even larger than the q_D_ value of 2 in Constellation I.

These challenges can be eliminated by the following explanation. We know that the figure–ground difference becomes more or less apparent in each constellation. As mentioned earlier, it is phenomenally more vivid in Constellations I and II than in Constellations III and IV. With regard to the numerical results, we observe the following: The difference between figure and ground thresholds is generally smaller for objectively brighter patches than for darker patches within each constellation. This observation, however, is not sufficient to completely explain Table I. It remains unclear why the q_D_ values are smaller in Constellation III than in Constellation I despite the small difference in brightness, and why the q_L_ values in Constellations I to III are very similar despite considerable differences in brightness. This can be explained in the following way: The influence of the objective patch brightness on the difference between figure and ground thresholds is being modulated by an additional factor; and the more vivid the figure–ground difference appears, the stronger the difference between figure and ground threshold becomes for a patch of a given objective brightness.

This hypothesis can be verified by the following experiment. For example, choose the following constellations:
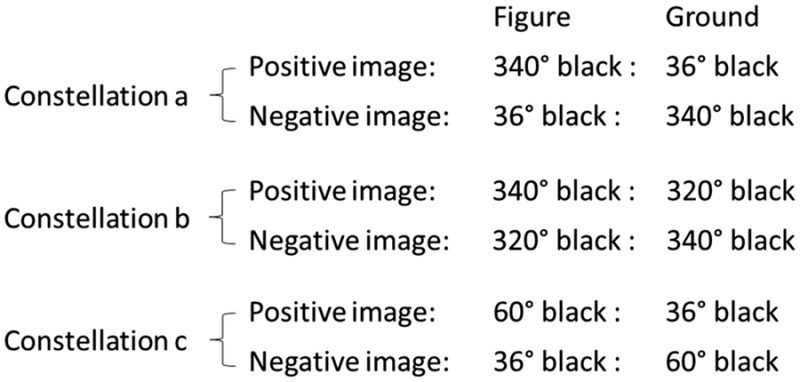


The darker patches in Constellations a and b and the lighter patches in Constellations a and c are of equal objective brightness (340° black and 36° black); however, as a result of the chosen brightness differences between the lighter and the darker patches, the positive and the negative images in Constellation a have to show a more pronounced figure and ground character as the positive image in Constellation b and the negative image in Constellation c.

Under these conditions, our aforementioned hypotheses are completely confirmed. The q_D_ values in Constellation a are considerably larger than those in Constellation b, and the q_L_ values in Constellation a are considerably larger than in Constellation c.^9^

According to the participants’ reports and our own observations, the emergence of the just noticeable color impression was different *on ground* than *on figure*; it became “suddenly clearly apparent” and could usually be localized immediately on the surface of the figure. On ground, in turn, it did not become immediately visible; moreover, the impression occured that it appeared like *out of fog* or *out of great depth* and continued to move to the front. These different ways of appearance were more pronounced in Constellations I and II than in Constellations III and IV, and even more pronounced in Constellations I and II when the color stimulus was added to the darker of the two patches.

### Aspects for Interpretation

1. When examining different possible explanations, one could first consider effects of simultaneous contrast.

Since in all of our images the ground’s expansion is larger than the figure’s, the figure is exposed to a stronger effect of contrast than the ground. Consequently, any patch of a given objective brightness must appear darker when presented as the positive image of a figure (i.e., on a brighter ground) than when presented as ground of the corresponding negative image. A patch of a given objective brightness, however, that is being used as the figure of a negative image (i.e., with a darker ground), has to appear subjectively even brighter than the ground of the corresponding positive image. Therefore, the subjectively stronger *blackness* of a figure in a positive image is the result of adding more black; however, the subjectively stronger *whiteness* of a figure in a negative image is the result of larger “subjective white adding” (G.E. Müller^10^).

If one wanted to attribute a significant influence of contrast effects to the results—independent of specific theoretical views—one would have to expect opposite effects for positive and negative images. In both cases, however, the figure threshold was higher than the ground threshold; this held true when the figure of the positive image appeared blacker than the ground of the corresponding negative image, and when the figure of the negative image appeared whiter than the ground of the corresponding positive image.^11^

Our results cannot, or at least not in a decisive manner, be the results of contrast effects, since the bright-ground threshold was lower than the dark-figure threshold for participant Gr. in Constellation IV. In this case, the subjective differences in brightness of the patches in question (230° black and 257° black) were even larger than those caused by the contrast effects described in Constellation I.

One could try to technically eliminate the influence of contrast by using figure and ground patches of the same size. One could, for example, use the pattern suggested by Rubin (1920), which allows either seeing a white cross on black ground or a black cross on white ground. Such a pattern proved not to be useful for our study, however, because it is not possible to look at Rubin’s or similar patterns without experiencing switches in figure and ground.

1. Recently Koffka (1921) reported on color threshold experiments at the Nauheim Meeting of Natural Scientists. He investigated whether color thresholds are exclusively dependent on the brightness of the gray to which one would add a color stimulus. He came to a negative conclusion with regard to this question and claimed that color thresholds depend on the brightness-structure between the test patch and its surrounding. He summarized his findings as follows: “The stronger the difference in brightness-structure between figure and ground, the higher the color threshold, and the more difficult it is to identify a color structure” (p. 162).

Since a detailed elaboration of Koffka’s hypothesis has not been published yet, we will not discuss it any further at this point. However, we note that differences between figure and ground thresholds cannot be explained by differences in brightness-structure, since both thresholds were measured at equal brightness-structures.

1. Can our main results be explained by the assumption that the figure thresholds were measured under better *attention* conditions than the ground thresholds?

Without any doubt, a figure patch attracts more attention than a ground patch; that is, a figure is “more attended to” than a ground patch. In that sense, attention conditions were not even worse but were better for the figure, and still the figure thresholds were higher. One can counter the assumption that the figure would receive less attention than the ground because its higher salience would be perceived as disturbing and therefore participants might guide their attention toward the ground, in the following way: If a distraction of this kind, which the authors did not perceive during the experiment, then this would have to be true for both measures of figure and ground thresholds. In our experimental conditions, participants’ attention was focused either on the center part of the lower wing of the cross or on the lower gap. In both cases, the center of the figure appeared at the same distance in the periphery. This theoretically possible distraction then should have been equally strong in both cases.

1. The explanation of our results lies within the fundamental differences between figure and ground. Rubin (1920) already explicitly pointed out that *attention* cannot explain this difference satisfactorily. Differences in *clarity* can also not account for our findings, since the comparison (the contrast between figure and ground) refers to “objects that are experienced as two different entities,” which are “two very concrete, phenomenologically real entities” (Köhler, 1920).

Following the characteristic phenomenal differences between figure and ground (the figure appears to be more vivid, solid, firm, to have a stronger thingness^12^ and can be located more precisely than the ground), we have to assume that the material correlates of the psychophysical impression of the figure are different from those of the ground. Rubin (1920) refrained from a psychophysical explanation of his results. However, Köhler (1920, p. 207) explained that the figure needs to be more vivid than the ground; he ascribes a higher psychophysical energy density to the figure compared with the ground, which shapes the correlate of the overall impression. The energy is more condensed in the figure, while the ground has a lower density at the given place.

Looking at our results from this vantage point, one could assume that the figure of a given objective brightness in our experiments corresponds to a “more vivid psychophysical event” than a ground of objectively equal brightness.

What happens phenomenally at the time when the added light is recognized? The moment the colorless or colored patch becomes visible—either on the figure or the ground—a new figure appears in the field of view and the given patch becomes the ground for the new figure.

That is, in our study, the added light patch appears at times on the ground and at other times on the figure patch. The physiological process corresponding to the generation of a new figure needs to prevail over an already vivid and dense psychophysical event in one case, whereas in the other, it would only have to overcome a comparably diffuse event.

According to this concept, the differences in resistance of the existing psychophysical state against the formation of a new figure would explain the higher figure thresholds compared with the ground thresholds in our study.

Even though we deem our explanation sufficient for our results, it is too specific when interpreted in the context of other perceptual phenomena. That the figure threshold is higher than the ground threshold reflects a more general rule, namely the tendency to form simple and preferably unambiguous Gestalt laws—Wertheimer’s (1920) principle of “good Gestalt.” This principle rules the so called color *alignment* impression—which has recently been described systematically (following Gestalt-psychological) aspects by Fuchs (1923)—which also plays a decisive role in out experiments. Since a color inhomogeneity limits the conciseness of our figures, the principle to appear as homogenous as possible comes into play; this explains a higher color threshold for the figure compared with equally bright ground patches. From this vantage point, the aforementioned *resistance* is simply a resistance against a change in the psychophysical events, which threatens the conciseness of the figure.

The former explanation implies that the figure threshold does not necessarily always have to be higher than the ground threshold. For example, if a given patch which only stands in light contrast to its surrounding and therefore only has weak figure character, gain stronger contrast, and figure character by adding more color to the whole patch, then it seems plausible that the figure threshold could be lower than the ground threshold in a different experimental setup. Similarly, the figure threshold should become more refined, if under partial coloring the figure increased in conciseness. This is supported by facts which have been studied in other areas and which were reported by Gelb (Gelb, 1922).

The facts presented here once more indicate how threshold values of a patch depend on its “Gestalt” character. This implies a methodological demand to always take this principle into account.

(Submitted January 10 1923)

## Supplementary Material

Supplementary material

## Notes

1 Translator’s note: This version of the manuscript has been shortened from the original. We have kept all details of methodology and results for their main experiment; most of the text that was edited out was in their discussion section, much of which was concerned with details of other research on color thresholds.

2 Translator’s note: The rotating disk referred to is of the type commonly used in color experiments of the time; color mixture was achieved by varying the proportions of individual colors in radial segments of a rapidly rotating disk. In the descriptions below on the creation of gray scale, degrees correspond to lightnesses that would be produced on such a disk.

3 *Figure* and *ground* are referred to in the sense of Rubin (1920).

4 An expert produced a set of color neutral photographs. The wet photographs were strapped on a stiff piece of cardboard and then treated with chalky paper in order to completely remove the shine from the photo paper. All dried images were equally matte.

5 Translator’s note: The colors were produced with the tools available at the time, and Tuchschwarz refers to a product name.

6 Translator’s note: 16 Normalkerzen is equivalent to approximately 14.7 candela.

7 To exclude a potential influence by the sound generated while moving the lamp on the participants, the experimenter generated a loud sound by foot scraping.

8 Since the angular position of the cardboard to trajectory of the lamp remained constant, we calculated the light units on the basis that the light intensity of a surface is the inverse of its squared distance to the light source.

9 An extensive description of such an experiment, which was conducted under slightly different technical conditions and theoretical context, shall be delivered at a later occasion. (Translator’s note: A similar follow-up experiment was reported by Granit in 1924 (Granit, R. (1924). Die Bedeutung von Figur und Grund für bei unveränderter Schwarz-Induktion bestimmten Helligkeitsschwellen). Scandinavisches Archiv Fur Physiologie*, 45*, 43–57. doi:10.1111/j.1748-1716.1924.tb00132.x

10 Translator’s note: Complete reference is not given.

11 It is also impossible to explain our results by the assumption that the figure’s brightness of a positive image was lower than the brightness of so called *critical grey* (compare p. 16). This assumption is incompatible with Constellation IV, since the *critical grey* corresponds to a brightness that is being referred to as black in everyday language and the patches in configuration IV were of *medium* brightness.

12 Translator’s note: “Dingcharakter”

13 A note on the references: The translators searched for accurate references that comply with current citation standards. However, at times, the citations in the original text were ambiguous (e.g., just author and no title or year). We tried to update those to the best of our knowledge.
