# Supplementary material for: Threshold Differences on Figure and Ground: Gelb and Granit (1923)
Source: Iperception. 2017 Jan 1;8(1):2041669516685722. doi: 10.1177/2041669516685722 (PMC5330600; doi:10.1177/2041669516685722)
Supplement: Supplementary material [file GG_Full_Translation.pdf]

Psychological studies of color
Edited by Adhémar Gelb

I.

The role of “figure” and “ground” for color thresholds.
(First report)

by
Adhémar Gelb and Ragnar Granit
(Helsinki)

Following convention, the color threshold is the amount of a color stimulus that, when mixed with a given gray field of a certain brightness, is just sufficient to reveal the color. Various studies of color thresholds have tested the dependency of threshold values on the brightness of the given gray fields and on the color stimuli that are combined with them. The purpose of those investigations was to clarify issues of color theory, e.g., relations between achromatic and chromatic visual processes[1].

To clarify the problem of interest, we first need to understand how previous studies theoretically evaluated a given grey patch to which color stimuli were added; specifically, how important it was for threshold values. Questions with regard to color threshold have been exclusively questions of brightness; stimuli have been presented in a certain part of the visual field and triggered a sensation using greyscale. The influence of the patch’s objective brightness, its brightness contrast with the background, or thresholds for two patches that were objectively of different but subjectively of the same brightness have been tested.

A brief consideration demonstrates that the current explanations are unsatisfactory.

Imagine the following threshold experiment: In one case, the grey patch (to which the color is to be added) is presented as a homogenous plane covering the entire field of view; in the other case, the patch is presented with the same (objective and subjective) brightness, but as a ring shape on a rotating disk[2]. Apart from questions regarding brightness and expanse, there is a fundamental difference between the two patches. The ring shape appears as a self-contained unit that delimits itself from the inside and the outside: the ring stands out as a “figure” from a brighter or darker “ground”.[3] In turn, the homogenous patch, which covers the entire field of view, appears evenly and uniform in general and does not, or at least in the same sense, appear as a “figure”; it is missing a brightness difference, without which seeing a figure in its actual sense is not possible.

---

[1] A note on the translation: Our aim in this translation was to provide a document that conveys the intended authors’ meaning.

[2] *Translator’s note: the rotating disk referred to is of the type commonly used in color experiments of the time; color mixture was achieved by varying the proportions of individual colors in radial segments of a rapidly rotating disk. In the descriptions below on the creation of grayscale, degrees correspond to lightnesses that would be produced on such a disk.*

[3] "Figure" and "ground” are referred to in the sense of Rubin (1920).

These examples may seem extreme. However, the characteristic differences remain, although not always as pronounced, under similar conditions: A grey patch formed by an entire rotating disk is – again independent from brightness and expanse -- phenomenally different than one that has the shape of a ring on a rotating disk. In the latter case, the surrounding of the ring (figure) has a distinctive ground character; in the former it does not. Likewise, a ring that does not differ strongly from the background is phenomenally a different patch than a ring with the same brightness that stands out from the background: in the latter case the grey patch is a "much better figure" and its background has a "clear ground character". Generally speaking, any grey patch selected for threshold studies can be described as either having more figure or more ground features.

Results reported by Rubin (1920) and elaborations by Köhler (1920) highlight the fundamental importance of figure-ground differentiation. This difference is not only phenomenological in nature, but, as Rubin showed in a series of experiments, two objectively identical patches exert fundamentally different psychophysical effects depending on whether they are experienced as figure or as ground. Rubin studied effects with regard to recognition, but also made observations about colors in relation to figure-ground effects (see Rubin, 1920, Figure 6).

Since the grey patch presented in studies on color thresholds has either more or less ground character, the question arises as to whether the color thresholds themselves vary depending on whether the grey patch has more features of ground or of figure. If dependencies between the just noticeable color impression and figure-ground experiences exist, these should have a significant impact on color theory. The specific question of the present study is: Is the color threshold (in a grey patch of a certain objective brightness) for a given intensity of a color stimulus different if the patch appears either as "figure" or as "ground"?

Methods and procedure

We used photographic techniques to produce the grey patches to which we added the color stimuli.[4] We used images showing a figure resembling a Maltese cross of about 5 cm diameter on a ground of about 15 cm diameter (see Figure 1). The size of each of the four wings of the cross and each of the four gaps corresponded to an eighth of a circle. The cross – the figure – was either darker or lighter than the ground. We will refer to images with a darker figure as "positive images" and to images with a lighter figure as "negative images".

Figure 1 about here

We used four different positive images, which varied in the brightness of figure and ground as well as in the brightness between figure and ground. Each of the four positive images had a corresponding negative image: For example, if the brightness of the figure was a mix of 340° black + 20° white produced on a disk and the brightness of the ground was 36° black + 324° white, the figure in the corresponding negative images was 36° black + 324° white and the ground was 340° black + 20° white.

[4] An expert produced a set of color neutral photographs. The wet photographs were strapped on a stiff piece of cardboard and then treated with chalky paper in order to completely remove the shine from the photo paper. All dried images were equally matte.

We derived an equation between each patch and a reference disk consisting of black (Tuchschwarz[5]) and white (barium white) sectors, to identify the brightness of the different patches. The reference disk and the patches were placed next to each other were observed from behind a screen with a hole at an appropriate distance, allowing observers to see only a small fraction of the reference disk and the patch. Brightness was considered as equal if the hole in the screen appeared to be of a completely homogenous, color neutral quality.

The following table provides a description of the different levels of brightness used in the study. Constellations I-IV refer to the the four different positive images and corresponding negative images and describe the relation of figure to ground brightness for both positive and negative images. For the sake of brevity, only the black proportions are shown.

		Figure	Ground
Constellation I	Positive image:	340° black :	36° black
	Negative image:	36° black :	340° black
Constellation II	Positive image:	337° black :	135° black
	Negative image:	135° black :	337° black
Constellation III	Positive image:	334° black :	260° black
	Negative image:	260° black :	334° black
Constellation IV	Positive image:	257° black :	230° black
	Negative image:	230° black :	257° black

There were the following descriptive differences between these four constellations: In constellation I, the difference between figure and ground appeared phenomenally the strongest, in constellation IV the weakest. Constellation II and III were in the middle, however constellation II was more similar to constellation I, and constellation III was more similar to constellation IV.

In order to investigate how the color threshold changes when the color stimulus is mixed with either a figure or a ground of a certain brightness, thresholds were determined for the figure (ground) of a positive image and then for the ground (figure) of the corresponding negative image. The values attained were then compared to each other.

The color thresholds were determined for a defined area of 3 mm diameter located as shown in Figure 1 on figure and ground [the two small circles]. The experiment used the following procedure.

The observer looked at the photograph which was placed in about 1 m distance, frontoparallel, and at eye-level. He looked through a monocular tube which only allowed viewing figure and ground, where the ground appeared to expand in all directions. A fairly large covering glass was placed in front of the tube-opening facing the image; its [the glass's] location was set so that a fraction of colored light coming from an adjustable lightsource at the side was mirrored into the observer's eye. That is, the color stimulus was mixed to the figure or ground by adding the colored light to the colorless light emitted by the image.

---

[5] *Translator's note: The colors were produced with the tools available at the time, and Tuchschwarz refers to a product name. It is unclear what the exact properties of these colors were.*

Figure 2 illustrates the essential details of the experimental setup.

Figure 2 about here

An electric lamp of 16 Normalkerzen[6] [Light source 1] was placed on a slider and could be moved easily and without noise.[7] A fraction of the light was reflected on a dark screen with a 3 mm opening by a white piece of cardboard which was placed in a constant position at an angle of the lamp's moving trajectory. A gelatine light filter was placed in front of the opening on the side facing the covering glass. The photograph was illuminated by a constant lightsource (Light source 2) invisible to to the observer and was of course placed at the same distance from the covering glass as the opening.

The intensity of the colored light was gradually decreased by changing the distance of the lamp to the reflecting cardboard; the distance of the lamp to the cardboard could be measured on a scale from 0-100 cm with a precision of 1 mm. Although this procedure was not sufficiently exact for our lamp (which radiated in all directions), we still used this method since we were only interested in the relative comparison between figure and ground thresholds and not in the numerical values [of the thresholds].

The experiments were conducted in a semi-darkened room. When the experimenter called "now", the observer, whose head was fixated by a chin-rest, was to look at the location of color stimulus (the center of the lower crosswing or the center of the right lower gap). The observer moved the light on the slider and then signalledto the experimenter the moment a true color impression became just visible. Participants were specifically instructed to only signal when they saw a color impression and not if a colorless or an unspecified spot became visible.

For each threshold measurement to be under the same adaptation conditions, the observer had to look into a semi-dark corner made out of two pieces of dark cardboard immediately after the end of each trial (see Figure 2).

To be able to safely compare the figure thresholds to the ground thresholds, color thresholds for each of the four constellations were identified in a single session. To avoid potential influence of practice or fatigue on the results, we completed the threshold measurements within each constellation in the following order: If the color stimulus was mixed to the dark patch, the threshold was first measured for the figure then for the ground; If the color stimulus was mixed to the light patch, the thresholds were measured in inverse order.

We only used the ascending method: Beginning at a position of the lamp at which no color was perceived, the lamp was gradually moved closer to the reflecting cardboard and the position was identified at which the color was first recognized (The sliding/moving of the lamp followed a mean speed which varied from case to case). Initially we also used the descending method, however, we decided to discard this method since it was subjectively felt to be unreliable and objectively lead to larger variance in the data.

As we were – at least initially – not interested in studying color thresholds for different colors, we almost exclusively used red light. In addition, we ran experiments using green light, but more for control

[6] *Translator's note: 16 Normalkerzen is equivalent to approximately 14.7 candela.*

[7] To exclude a potential influence by the sound generated while moving the lamp on the participants, the experimenter generated a loud sound by foot scraping.

purposes and only using constellation I. We used gelatine light filter#25 (“scarlet”) and #14 (“methyl green and picric acid”) provided by Dr. Steeg & Reuter in Homburg v.d.H. The filtered light, especially by the red filter, was tested with an objective spectrum and found to be very pure.

In addition to us (Gb. and Gr.) the following gentlemen participated in the study: Mr. stud. phil. Wenzel (Wz.), Mr. stud. phil. Schriever (Schr.), Mr. stud. phil. Steuerwald (St.), Mr. stud. phil. Greb (Grb.), and Mr. Wingenbach (Wgb.). None of the gentlemen, whom we would like to thank for their participation, were familiar with the research question.

Results

Table I shows the threshold values for red, consisting of mean values from five single trials. Prior to these trials, participants completed three to five trials (not reported in the table) in which participants practiced the task (and were acclimated to the procedure).

The table should be read as follows:

Rows: The first row contains the distance of the lamp to the reflective cardboard in cm (compare with the sketch in Figure 2). The second row contains the corresponding mean variations (m.V.); in the third row we report the values of the first column in the corresponding light units (L.E.), by setting the light intensity of the cardboard at 1 when the lamp was a distance of 1 m from the reflecting cardboard[8]. That is, the light intensity of the cardboard served as a reference point for the threshold values.

Columns: Columns labeled D.F. (“dark figure”) refer to thresholds for constellations with a darker figure and a lighter ground, i.e. figures with 340° black, 337° black, 334° black, and 257° black. D.G. (“dark ground”) refers to thresholds with a corresponding darker ground. The third row, labeled Q_d reports the proportion of the figure and ground threshold values.

Columns labeled L.F. (“light figure”) and L.G. (“light ground”) refer to threshold values for cases in which the lighter figure and ground, i.e. patches with 36° black, 135° black, 260° black, and 230° black; Q_L refers to the corresponding proportions.

Table I here

Table I. (color: red)

The following conclusion can be drawn from Table I:

(1) First, confirming our previous results, adding a color stimulus to a whiter patch led to higher color thresholds than adding a color stimulus to a blacker one. If one finds a deviation from this rule with participant Wz., one has to consider that the chosen experimental paradigm only allows for a comparison between thresholds within a constellation. In addition, practice and experience seem to have played a role with this participant.

---

[8] Since the angular position of the cardboard to trajectory of the lamp remained constant, we calculated the light units on the basis that the light intensity of a surface is the inverse of its squared distance to the lightsource.

(2) Second, the main findings are: The color threshold of any given patch of objectively same brightness varied depending on whether the respective field was seen either as figure or as ground. The figure threshold was higher than the ground threshold, independent from the objective brightness (all q_d and q_h values are bigger than 1).

(3) Third, this observation was more pronounced for the darker patch compared to the lighter patch within each constellation: The q_d value of a constellation was, on average, larger than the corresponding q_h value. The only exception was the values in constellation III and IV of participant Gr.

(4) Fourth, a closer look at Table I reveals that the influence of figure-ground on the color threshold varied over different constellations.

Compare the q_d and q_L values for constellation I-III. Despite the relatively small differences in objective brightness of constellation I and III (340° black in constellation I vs 334° black in constellation III), the q_L values, for instance, in constellation I (participants Gb., Gr., Wz.) are noticeably smaller than in constellation III. One could assume that the lower q_L values in constellation III are the result of a brighter patch than in constellation I. This assumption could be further supported by the notion that the subjective brightness of the patch in constellation III is lower than in constellation I as a result of adding less black to the color mix. However, this would hardly be in accordance with the observation that the participant Gb.'s q_L values in constellation I (1.13) are only marginally smaller than the q_L values in constellation III (1.17), although constellation I (36° black) is objectively significantly brighter than constellation III (260° black). In addition, the stronger contrast in brightness in constellation I increases the the subjective brightness of the field even more. Further, this would not be in accordance with the q_L value of 1.22 (participant Wz.) being even larger than as the q_L value of 1.1 in constellation III (We have to ignore the the q_L value of 1.76 of participant Gr. in constellation III at this point as he had been identified as an outlier earlier). Equally hard to understand would be why the q_L values in constellation I (participant Gb., Gr., Wz.) almost completely match the q_L values in constellation II, although the corresponding patches still differ considerably in objective brightness (36° black in constellation I and 135° black in constellation II). It would also remain unclear why the q_D values of participants Gb. and Wz. in constellation II are smaller than in constellation I, although the proportion of white is comparable in both cases (340° black and 337° black), and why the q_D values of 2.83 (participant Gr.) in constellation II are even larger than the q_D value of 2 in constellation I.

These challenges can be eliminated by the following explanation. We know that the figure-ground difference becomes more or less apparent in each constellation. As previously mentioned, it is phenomenally more vivid in constellations I and II than in constellations III and IV. With regard to the numerical results we observe the following: The difference between figure and ground thresholds is generally smaller for objectively brighter patches than for darker patches within each constellation. This observation, however, is not sufficient to completely explain Table I. It remains unclear why the q_D values are smaller in constellation III than in constellation I despite the small difference in brightness, and why the q_L values in constellation I – III are very similar despite considerable differences in brightness. This can be explained in the following way: The influence of the objective patch brightness on the difference between figure and ground thresholds is being modulated by an additional factor: The more vivid the figure-ground difference appears, the the stronger the difference between figure and ground threshold becomes for a patch of a given objective brightness.

This hypothesis can be verified by the following experiment. For example, choose the following constellations:

		Figure	Ground
Constellation a	Positive image:	340° black :	36° black
	Negative image:	36° black :	340° black
Constellation b	Positive image:	340° black :	320° black
	Negative image:	320° black :	340° black
Constellation c	Positive image:	60° black :	36° black
	Negative image:	36° black :	60° black

The darker patches in constellations a and b and the lighter patches in constellations a and c are of equal objective brightness (340° black and 36° black); however, as a result of the chosen brightness differences between the lighter and the darker patches, the positive and the negative images in constellation a have to show a more pronounced figure and ground character as the positive image in constellation b and the negative image in constellation c.

Under these conditions, our aforementioned hypotheses is completely confirmed. The q_D values in constellation a are considerably larger than those in constellation b and the q_L values in constellation a are considerably larger than in constellation c[9].

(5) Given our results, constellation IV is particularly interesting. We will take a closer look at the different threshold values for the four participants.

For participants' Gb. and Schr., the bright-ground threshold for a patch of 230° black (7.67 and 5.2) was larger than the dark-ground threshold for a patch of 257° black (4.48 and 3.87) in this, as well as in the other constellations. That is, the influence of the patch's brightness on the threshold was stronger than the figure-ground factor in this case.

In the case of participant Gr., the bright-ground threshold (2.8) was smaller than the the dark-figure threshold (3.27)[10]. For this participant, adding red light to a ground patch of 230° black resulted in a lower threshold than adding red light to a figure patch of 257° black. In other words: In this case the influence of the figure-ground factor predominated the brightness of the patch (Although it has to be mentioned that for this participant, the bright-ground threshold was almost the same as the dark-ground threshold).

---

[9] An extensive description of such an experiment, which was conducted under slightly different technical conditions and theoretical context, shall be delivered at a later occasion. *(Translator's note: a similar follow-up experiment was reported by Granit in 1924 (Granit, R. (1924). Die Bedeutung von Figur und Grund für bei unveränderter Schwarz-Induktion bestimmten Helligkeitsschwellen). Scandinavisches Archiv Fur Physiologie, 45(1), 43-57. doi:10.1111/j.1748-1716.1924.tb00132.x))*

[10] A similar result was observed in constellation III for this participant; however, we cannot consider these results because of the exclusion of the q_L value of 1.76.

Participant Wz.’s bright-ground threshold (1.88) corresponded to his dark-figure threshold (1.89). The effect of the figure-ground factor compensated for the effects of the objective brightness of the patches.

We also determined the color threshold for green using constellation I and testing ourselves. This study showed the same main result as the threshold determination for red; the numerical results are depicted in Table II:

Table II here

Let us focus on the phenomenal aspects. The color stimulus was initially perceived as “something bright” or as a patch of a nondescript color, especially when it was added to an objectively dark patch; soon after that the specific color became visible.

According to the participants’ reports and our own observations, the emergence of the just noticeable color impression was different “on ground” than “on figure”; it became “suddenly clearly apparent” and could usually be localized immediately on the surface of the figure. On ground, in turn, it did not become immediately visible; moreover, the impression occurred that it appeared like “out of fog” or “out of great depth” and continued to move to the front. These different ways of appearance were more pronounced in constellations I and II than in constellations III and IV, and even more pronounced in constellations I and II when the color stimulus was added to the darker of the two patches.

Control observations

The reported numerical results were confirmed in a qualitative control study using constellation IV. Apparatus and procedure were identical to the previous study, with the exception that we used a larger opening of 3 cm diameter on the screen (see Figure 2), as opposed to 3 mm in the previous study. The position of the opening was chosen so that the colored disk of about 3 cm diameter was visible when the stimulus appeared well above threshold. The center of the disk was the same as the center of the cross.

We identified the amount of color stimulus that needed to be added for its (color) quality to be just recognizable on any place of the image. The observer, who was looking at the image with an un-fixated eye, indicated where (figure or ground) he first observed the color on the image. The experiments were completed with the positive and the negative image of constellation IV. The color of the added light was red.

Constellation IV, positive image. When discussing the results of Table I, we saw that in constellation IV different patterns emerged for different participants. Since, as we shall see, the results varied from individual to individual, it is useful to discuss participants’ results individually.

Participant Gb. This participant detected the color red on the (darker) figure (257° black) at a slightly lower intensity of the added colored light than on the (brighter) ground (230° black). Similarly, we observed that this participant’s threshold values were lower for the figure than for the ground in the initial experiment.

Participant Gr., however, detected the color red first on the ground, and the intensity of the added light needed to be increased slightly for the color to become visible for him on the figure. This participant's results were similar throughout Table I (constellation IV).

The participants St. and Wgb., whom we recruited as new observers, reacted like participant Gr.

Participant Wz. recognized the color of the additional stimulus at about the same point on figure and ground. In four consecutive single trials, which we randomly picked from the sample, these values were practically identical. This result also confirms the observations in Table I from this participant.

Particpant Wz.'s responses were in full accordance with the previous results, when we added light with an intensity far above threshold and then asked him if he saw the color red more clearly on the figure or the ground. The behavior that the participant showed was characteristic; he said: "red is clearer on the figure, - no, no – on the ground – no, on the figure or – maybe the same overall". These repeatedly stated comments are indicative of a competition or a repeated switching of impression. Participant Gb. confirmed this for stimuli far above threshold.

In fact, we observed that the results are aligned completely with the findings in Table I. This alignment is even more valuable, since the data was recorded much later than the results of Table I.

We gained certainty that our findings were neither the result of any random factors nor influenced by suggestions through the experiments, using the negative image in constellation IV, which were conducted following the same procedure: In this experiment, the ground was darker than the figure and consequently brightness and figure-ground worked in the same direction. All participants identified the color stimulus at a lower light intensity than on the figure.

Observations of the negative image in constellation I: At this point it is worth mentioning another observation that confirms our main results, which we were able to make with the negative image in constellation I. Once more, we used the larger opening in the screen and added red light far above threshold. Of course, all participants experienced red on the black ground as much more intense than on the white figure. This difference in intensity was so large that the red color on the black ground became a distinguishable closed unit setting itself apart from the rest of the patch. The white cross lost much of its figure character and became closer to the ground character. (This happened all by itself and not only after deliberately highlighting the cross).

The following observation is essential: As soon as the tilted red cross dominated the figure, the white cross highlighted the red color more; however, as soon as the white cross was seen as the figure, its color appeared paler.

When we switched to experiments in which we deliberately alternated between highlighting the straight white cross and the small tilted red cross as the figure, this phenomenon became even more apparent: the red color faded noticeably on the white cross if it was singled out as the figure, and it increased in intensity if the tilted red cross was singled out. The participants St., Wgb., and Grb. confirmed this observation.

In this context we refer to a similar observation by Rubin (1920, Figure 6), who used patterns on which white crosses on black ground and black crosses on white ground became alternatingly visible. He then

let a shadow fall on the white part of the pattern and noted that the shadow became clearer if the dark parts appeared as figures and the white parts as ground than in the inverse case.

Aspects for interpretation

1. When examining different possible explanations, one could first consider effects of simultaneous contrast.

Since in all of our images the ground's expansion is larger than the figure's, the figure is exposed to a stronger effect of contrast than the ground. Consequently, any patch of a given objective brightness must appear darker when presented as the positive image of a figure (i.e., on a brighter ground) than when presented as ground of the corresponding negative image. A patch of a given objective brightness, however, that is being used as the figure of a negative image (i.e., with a darker ground), has to appear subjectively even brighter than the ground of the corresponding positive image. Therefore, the subjectively stronger "blackness" of a figure in a positive image is the result of adding more black; however, the subjectively stronger "whiteness" of a figure in a negative image is the result of larger "subjective white adding" (G.E. Müller[11]).

If one wanted to attribute a significant influence of contrast effects to the results – independent of specific theoretical views – one would have to expect opposite effects for positive and negative images. In both cases, however, the figure threshold was higher than the ground threshold; this held true when the figure of the positive image appeared blacker than the ground of the corresponding negative image, and when the figure of the negative image appeared whiter than the ground of the corresponding positive image.[12]

Our results cannot, or at least not in a decisive manner, be the results of contrast effects, since the bright-ground threshold was lower than the dark-figure threshold for participant Gr. in constellation IV. In this case the subjective differences in brightness of the patches in question (230° black and 257° black) were even larger than those caused by the contrast effects described in constellation I.

Finally, we refer to the results of the control observation, which also cannot be explained by contrast effects.

One could try to technically eliminate the influence of contrast by using figure and ground patches of the same size. One could, for example, use the pattern suggested by Rubin (1920), which allows either seeing a white cross on black ground or a black cross on white ground. Such a pattern proved not to be useful for our study, however, because it is not possible to look at Rubin's or similar patterns without experiencing switches in figure and ground.

2. Recently Koffka (1921) reported on color threshold experiments at the Nauheim Meeting of Natural Scientists. He investigated whether color thresholds are exclusively dependent on the brightness of the grey to which one would add a color stimulus. He came to a negative conclusion with regard to this

[11] *Translator's note: complete reference is not given.*

[12] It is also impossible to explain our results by the assumption that the figure's brightness of a positive image was lower than the brightness of so called "critical grey" (compare p. 16). This assumption is incompatible with constellation IV, since the "critical grey" corresponds to a brightness that is being referred to as black in everyday language and the patches in configuration IV were of "medium" brightness.

question and claimed that color thresholds depend on the brightness-structure between the test patch its surrounding. He summarized his findings as follows: ”The stronger the difference in brightness-structure between figure and ground, the higher the color threshold, and the more difficult it is to identify a color structure.“ (p. 162).

Since a detailed elaboration of Koffka‘s hypothesis has not been published yet, we will not discuss it any further at this point. However, we note that differences between figure and ground thresholds can not be explained by differences in brightness-structure, since both thresholds were measured at equal brightness-structures.

3. Can our main results be explained by the assumption that the figure thresholds were measured under better ”attention“ conditions than the ground thresholds?

Without any doubt a figure patch attracts more attention than a ground patch; i.e., a figure is ”more attended to“ than a ground patch. In that sense, attention conditions were not even not worse but were better for the figure, and still the figure thresholds were higher. One can counter the assumption that the figure would receive less attention than the ground because its higher salience would be perceived as disturbing and therefore participants might guide their attention towards the ground, in the following way: If a distraction of this kind, which the authors did not perceive during the experiment, then this would have to be true for both measures of figure and ground thresholds. In our experimental conditions, participants’ attention was focused either on the center part of the lower wing of the cross or on the lower gap. In both cases the center of the figure appeared at the same distance in the periphery. This theoretically possible distraction then should have been equally strong in both cases.

4. The explanation of our results lies within the fundamental differences between figure and ground. Rubin (1920) already explicitly pointed out that ”attention” cannot explain this difference satisfactorily. Differences in ”clarity“ can also not account for our findings, since the comparison (the contrast between figure and ground) refers to ”objects that are experienced as two different entities“, which are ”two very concrete, phenomenologically real entities“ (Köhler, 1920).

Following the characteristic phenomenal differences between figure and ground (the figure appears to be more vivid, solid, firm, to have a stronger thingness[13], and can be located more precisely than the ground), we have to assume that the material correlates of the psychophysical impression of the figure are different from those of the ground. Rubin (1920) refrained from a psychophysical explanation of his results. However, Köhler (1920, p. 207) explained that the figure needs to be more vivid than the ground; he ascribes a higher psychophysical energy density to the figure compared to the ground, which shapes the correlate of the overall impression. The energy is more condensed in the figure, while the ground has a lower density at the given place.

Looking at our results from this vantage point, one could assume that the figure of a given objective brightness in our experiments corresponds to a “more vivid psychophysical event” than a ground of objectively equal brightness.

What happens phenomenally at the time when the added light is recognized? The moment the colorless or colored patch becomes visible – either on the figure or the ground – a new figure appears in the field of view and the given patch becomes the ground for the new figure.

---

[13] *Translator’s note: “Dingcharakter”*

That is, in our study, the added light patch appears at times on the ground and at other times on the figure patch. The physiological process corresponding to the generation of a new figure needs to prevail over an already vivid and dense psychophysical event in one case, whereas in the other it would only have to overcome a comparably diffuse event.

According to this concept, the differences in resistance of the existing psychophysical state against the formation of a new figure would explain the higher figure thresholds compared to the ground thresholds in our study.

The fact that the results of our control observations are in line with this reasoning toned not be explicated, since the difference between the main and the control study was the surface size of the added color stimulus.

Even though we deem our explanation sufficient for our results, it is too specific when interpreted in connection to many other perceptual phenomena. That that the figure threshold is higher than the ground threshold reflects a more general rule, namely the tendency to form simple and and preferably unambiguous Gestalt laws – Wertheimer's (1920) principle of "good Gestalt". This principle rules the so called color "alignment" impression – which has recently been described systematically (following Gestalt-psychological) aspects by Fuchs (1923) - which also plays a decisive role in out experiments. Since a color inhomogeneity limits the conciseness of our figures, the principle to appear as homogenous as possible comes into play; this explains a higher color threshold for the figure compared to equally bright ground patches. From this vantage point the aforementioned "resistance" is simply a resistance against a change in the psychophysical events, which threatens the conciseness of the figure.

The former explanation implies that the figure threshold does not necessarily always have to be higher than the ground threshold. For example, if a given patch which only stands in light contrast to its surrounding and therefore only has weak figure character, gain stronger contrast and figure character by adding more color to the whole patch, then it seems plausible that the figure threshold could be lower than the ground threshold in a different experimental setup. Similarly, the figure threshold should become more refined, if under partial coloring the figure increased in conciseness. This is supported by facts which have been studied in other areas and which were reported by Gelb (Gelb, 1922).

The facts presented here once more indicate how threshold values of a patch depend on its "Gestalt" character. This implies a methodological demand to always take this principle into account.

Application of our views to results of other studies

1. Révész (1907a) provides extensive research on the behavior of color thresholds "first under increasing intensity of the given white stimulus and second, under increasing strength of the given black stimulus."

In his first series of experiments he added spectral color light to a patch of 4 mm diameter und increased the white value of the patch stepwise from 0° to 360°. The surrounding of the patch was always black. He found that the color threshold increased with the white value. The lowest threshold was to be found at 0°, or rather when brightness of the patch was close to black.

In his studies with increasing strength of black stimulation, Révész used a patch (a field of 4 mm diameter) that could subjectively appear darker than the 0° white patch from in the first experiments by

using simultaneous brightness contrast. A stepwise increased exposure of the surrounding induced increasingly stronger black stimuli.

Such experiments show that a weak light exposure in the surround would allow for an even finer grained threshold than in the first series of experiments with 0° white and that the color threshold continued to increase stepwise. Révész concluded that there was a "critical grey" where the minimum color threshold lies. This critical grey was reached by illuminating (with colored light) the surround of a black stimulus that was just enough to compensate for the white value that was added. In this case, "the minimal sum of intensities of the white and black stimulation existing in our center of vision have been reached."

According to Stumpf (1917) this definition is theoretical in nature. The tables of the first of Révész's experiments show that empirically that smallest color thresholds are reached if the added grey is close to the surrounding black. Furthermore, in the second series of experiments the smallest color threshold was observed at an "almost black surrounding, i.e. with minimal addition of black".

Phrased differently, Révész's observations become relevant for our results: The lowest color threshold was when the observed patch was in a) no or b) insufficient contrast to the figure.

The notion given on p. 1 can contribute to understanding: If the small patch lacks figural features (case a), such as in Révész's study, the observer will see a homogenous patch filling the whole field of view. In this case adding a very small amount of color to the patch is enough to make it "stand out as a figure". If the small patch does not form a vivid enough figure (case b), the small patch becomes a figure of little conciseness; this is the case in Révész's second series of experiments, when the difference in brightness between the patch and the surrounding is very small. In this case the color threshold may be even smaller than in case (a) because according to the principle of Prägnanz the "worse" figure now has the tendency to become more concise. Under these conditions the color threshold is very low. These deliberations are supported by Stumpf's experiments, which found that that the color threshold is lowest if "the brightness of the added grey is identical or almost identical to the brightness of the background." (A small deviation in brightness, according to Stumpf, might be optimal (as cited earlier, see p. 1))

Using the principle of Prägnanz may be sufficient to explain the minima of the thresholds found by Révész. However, can it also explain Révész's other results? Can we explain why the color threshold increased when the brightness of the patch itself or its surrounding was gradually increased, meaning that the patch seemed more like a figure even before any color was added?

Since Révész mixed the color to the whole figure (unlike in our experiment), a change in color homogeneity (as observed in our studies) could not cause the increase in color thresholds. Following the principle of Prägnanz, a figure tends to appear as one salient color, i.e. either without any color tone at all or "thoroughly colored"; That is, a figure will "resist" to a change in its (perceived) color until the amount of color added is sufficient to generate a new nearly salient tone. Therefore, color added to a colorless small figure patch only becomes recognizable if it "saliently colored" (This is in line with the phenomenological observations.) The larger the difference in brightness between the small patch and its surrounding in Révész's study, independent of whether it was due to a change in brightness of the figure or its surrounding, the more salient the figure and color character of the small patch became and the more it "resisted" the added color.

Given that the color threshold was lowest at the point of "critical grey" and increased from there independent of whether more white or black was mixed to the patch, Révész's interpretation becomes less convincing. In both cases it no longer possible to determine on how the proportion of black and white added to the patch affected the increase in the threshold values.[14]

At this point is not possible to assess whether Révész's explanations are valid but the theoretical implication which Révész assigned to the "critical grey" has to be questioned.

Révész used a "Farbenkreisel" in another study (Révész, 1909). He added a color stimulus to a ring with a brighter surrounding. Révész then identified the minimal color threshold at different levels of contrast. He found that "the absolute value of the color threshold increased with the surrounding's brightness; the stronger the contrast the higher the threshold values" (p. 359).

Révész did not expect these results because he compensated for the surrounding's increase in contrast by adding white to the ring so that the ring was at always at a "critical grey" level. Révész points out that "chromatic stimulation is strongest at the critical grey and since the critical grey usually has a similar intensity, color threshold levels should be independent from the amount of white stimuli added. Yet we still found a continuous increase in color thresholds with increasing amounts of white being added" (p. 359). Révész speculates that his results can be explained by the amount of white necessary to compensate for decreases in the effects of the colored light stimulus.

The question is whether this additional assumption is still necessary. Consider that by increasing the intensity of the contrasting patch, the ring's figure character became more and more apparent; this alone could explain the increased color thresholds (see p. 1 for an explanation).

3. In a third study Révész investigated color thresholds on two grey patches of objectively different brightness but by using brightness contrast subjectively appeared to be equally bright (Révész, 1907b). The goal was to identify "if the color threshold for both patches was the same when they appeared to be equally bright". Both grey patches consisted of rings with 1 cm diameter, one presented on a black (360° Tuchschwarz) and the other on a white background (360° barium white). Following Révész, the ring on the black background will be referred to as the D-field or D-ring and the ring on the white background as the B-field or B-ring. On the D-ring, Révész adjusted sectors at a constant 354° black and 6° white. A B-ring appeared to be equally bright to the D ring at 76° white.

After establishing equal brightness on the two rings, Révész identified the color threshold for the primary colors and found the color threshold to be much higher for the B-ring for all four colors. (Révész calculated the ratio of the color thresholds of the B and the D-field, which he refers to as the attenuation coefficient, and was largest for blue and smallest for yellow).

The colors were less recognizable when added to the B-ring compared to the D-ring although both rings were subjectively equally bright. Révész claims that this can only be explained the following way: "White triggers a process on the retina (in the cone cells), which strength is determined by physical light

---

[14] The argument holds true for the results of Angier (1907) which are the same as Révész's in principle. He found that adding white light either to the patch or its surround increased the threshold values. The threshold values increased in both cases, „although in the former the subjective brightness of the field increase the saturation of the color decreased; and in the latter the subjective brightness of the field decreased and saturation increased." (p.360).

intensity but is not modified by brightness contrast effects. Since the B-field emits stronger weight light than the D-field, this process is stronger in those areas of the retina that correspond to the B-field". (p.115)

Katona (1921) recently confirmed Révész's main result. He argues that the "antichromatic influence of white" (G.E. Müller) on the B-ring is stronger since the white sector is larger than the D-ring. This is independent of the perceived equal brightness, according to Katona, "since the brightness contrast causing the subjectively equal brightness plays out in a more central zone." (p.166)

We won't go into a detailed discussion of Katona's interpretation of his results. First, we aim to show that the core observation, the increase of the color threshold in the B-ring, can be explained by our results.

One ring containing 76° white on white ground and ring containing 6° white on black ring need to be produced on a rotating disk. When looking at these two rings under average daylight conditions they appear to be equally bright; However, the B-ring stands out as a clear figure from the ground, it is harder to see the D-ring as a closed ring. One "needs to almost search" for the ring (Prof. Schumann) or: "only parts of the ring can be seen clearly" (Gb., Wgb.); if one tries to look at one part more clearly, one usually loses sight of the other parts of the ring. In short, the B-ring has clear figure character and the D-ring has not. At times, one has the tendency to even see the surrounding of D-ring as a figure with a ring shaped hole in it.

Therefore, Révész's threshold studies confirm our observations (p. 1).

4. Before discussing further experimental evidence for our view, we want to point out one more specific result by Katona (1921) which also confirms our explanation.

In one series of experiment, Katona changed the white sector from trial to trial; in each trial a seemingly equally bright ring was investigated. In the first constellation, the D-ring consisted of 12° white, in the second constellation 6° white, and in the third 2° white. The white sectors in the B-ring on average had 135° white in the first constellation, 76° white in the second, and 50° white in the third.

The threshold increase in the B-ring was larger in the third constellation; The absolute value of the attenuation coefficient ε was larger in the third constellation than in the first. Kartona argues "that the white sectors in the D- and B-ring were at about 12° white in constellation I, 135° white in constellation II, and 50° white in constellation III. Here, the proposition that the increase of the antichromatic influence of white, […] is the stronger, the lower the initial proportion of white is. Increasing the amount of white for a patch of 2° white may have a very different increase in achromatic influence than adding the same or even larger amount of white to a patch of 12° white." (p.171)

Kantona's results fit well with our interpretation. Consider constellations I and III and compare them with regard to figure-ground differences.

Constellation I (12° white on the D-ring, 135° white on the B-ring): Both rings appear as a figure, one on darker and the other on brighter ground; the B-ring has stronger figure character.

Constellation III (2° white on the D-ring, 50° white on the B-ring): The B-ring is an excellent figure; the D-ring is barely visible and one sees an almost homogenous black surface.

From this description the phenomenal differences between the figure-ground experiences in constellation III is much larger than in constellation I. This alone could explain that the less fine grained thresholds in the B-ring (absolute value of the attenuation coefficient ε), are larger in constellation III then in constellation I.

5. A similar interpretation would account for results observed by Hermann (1913) in a continuation of Révész's studies. Hermann tested whether the attenuation coefficient ε "dependent on the brightness of the contrast-inducing bright ground". While the D-ring was kept at a constant 6° white, the ground for the subjectively equally bright B-ring was set to varying degrees of brightness by adding either 90°, 180°, 270°, or 360° of white. The attenuation coefficient of the colors studied increased with the brightness of the contrast inducing field. Following our interpretation, the attenuation coefficient increased the stronger the figure and color character of the B-ring.

6. Further a study reported by Koffka at the 1920 Conference of natural scientists. The intention of Koffka's study is not of interest here (also see p. 1). We will simply describe this study because it verifies our explanation five on p. 1 and at the same time contradicts previous explanations. Koffka placed a ring on a white background that only differed slightly from the white surrounding. He placed a D-ring of subjectively equal brightness on a black background next to the B-ring. That way, unlike in Révész's and Katona's studies, the role of the two rings were interchanged: now the D-ring had stronger figure character than the B-ring, and the color thresholds were the opposite of the results observed by Révész and Katona. The threshold values were now larger on the D-ring.

This observation, which is in line with our interpretation, cannot be explained with Révész' and Katona's interpretation. In Koffka's experiment the proportion of white is larger in the B-ring than in the D-ring – which made them appear to be equally bright. Therefore, the "antichromatic effect of white" is stronger in the B-ring. Consequently, a reduction of the threshold values in the B-ring cannot be explained by the weakening effect of white light.

We replicated Koffka's study in a slightly modified way and confirmed his results. The B-patch was a homogeneous white rotating disk (360° white), which consisted of three concentric white disks with different radii. The largest disk was set at the bottom, the intermediate disk on top of it, and the smallest disk was set on top, which had a radius 1 cm smaller than the medium disk. Close to it was a second rotating disk with the following configuration: The largest and the smallest disks were black, and the intermediate disk was white, creating a 1 cm wide ring (a D-patch) which was subjectively equally bright to the B-patch. The D-ring required 106° black to appear equal to the 0° black. Equal brightness was accomplished by gradually comparing the middle part of the B-patch.[15] Both disks were uniformly illuminated.

After equal brightness was accomplished colored paper disk was added to the D- ring and the B-patch (within an intermediate 1 cm wide ring). For example, 19° red was added to both patches. We used the dark-red, dark-blue, and the green paper of the Hering series. The observer had to determine whether the added color was more easily recognizable on the D-ring or on the B-patch.

---

[15] We confirm Révész's and Katona's claim that producing equal brightness is difficult. The equation is slightly different for individual observers, depending on whether the patches are compared simultaneously or gradually (after each other) .

With no exceptions, observers confirmed that the mixed colors were more easily recognizable on the 0° black B-ring than on the 106° black D-ring. (Some observers did not reach an agreement on the label of the observed color). Adding a larger proportion of colored paper to the D-ring compared to the B-ring did not change the results. Note the following example:

	B-ring = 360° white<br>D-ring = 254° white + 106° black	
In D-ring	In B-ring	Protocol/Observation[16]
19° red	19° red	D-ring appears white; B-ring clearly rose
20° blue	20° blue	D-ring appears white; B-ring faint blueish or greenish
44° blue	44° blue	Rings appear blue; color clearer in B-ring
60° green	50° green	Rings appear greenish; color much clearer in the B-ring
80° green	74° green	The same

7. Finally, we based on ongoing data collection, we intend to report that brightness thresholds are investigated under similar conditions as our reported color thresholds provide a comparable figure and ground experience, which allows to explain the results of several other studies (e.g., Blachowsky, 1913; Dittmers, 1920; Seffers, 1922).

Jaensch's (1920) studies on just noticeable brightening of a patch when changing the ground brightness fall into this line of reasoning. Jaensch claims to have proven the following principle: "Corresponding to the transformation studies, the just noticeable brightening is reached by adding the same amount of light, independent of whether the infield contrast is being brightened or darkened." (p.344). Dittmers (1920) could show that this observation was incorrect, based on his experiments. Jaensch only used the black and white surrounding. From this point of view it is easy to understand that the difference threshold is lowest when the figure and ground are equally bright. Under such conditions, the figure and ground conditions allow for easy threshold detection, since the conditions for establishing a new "figure" are best. The threshold increases when the ground brightness increases to white or decreases to black, since in both cases the inner field becomes a "figure".

(Submitted January 10 1923)

References[17]

Angier, R. (1907). Über den Einfluß des Helligkeitskontrastes auf Farbenschwellen. *Zeitschr. f. Sinnesphysiologie, 41*, 343-363.

Blachowsky, S. (1913). Studien über den Binnenkontrast. *Zeitschr. f. Sinnesphysiologie, 47*, 291-330.

---

[16] The protocol reflects the observations of participant Gb. Since these are typical, we only report those.

[17] A note on the references: The translators searched for accurate references that comply with current citation standards. However, at times the citations in the original text were ambiguous (e.g., just author and no title or year). We tried to update those to the best of our knowledge.

Dittmers, F. (1920). Über die Abhängigkeit der Unterschiedsschwelle für Helligkeiten von der antagonistischen Induktion. *Zeitschr. f. Sinnesphysiologie, 51*, 214-232.

Fuchs, W. (1923). Untersuchungen über das simultane Hintereinandersehen auf derselben Sehrichtung. *Zeitschrift für Psychologie, 91*, 145-235.

Gelb, A. (1922). *Grundfragen der Wahrnehmungspsychologie*. Paper presented at the IVV. Kongress fuer experimentelle Psychologie, Jena, Germany.

Hermann, J. (1913). Über die Fähigkeit des weißen Lichtes die Wirkung farbiger Lichtreize zu schwächen. *Zeitschr. f. Sinnesphysiologie*, 97-105.

Jaensch, E. (1920). Parallelgesetz über das Verhalten der Reizschwellen bei Kontrast und Transformation. *Zeitschrift für Psychologie, 83*, 342-354.

Katona, G. (1921). Experimentelle Beiträge zur Lehre von den Beziehungen zwischen den achromatischen und chromatischen Sehprozessen. *Zeitschr. f. Sinnesphysiologie, 53*, 145 and following.

Köhler, W. (1920). *Die physischen Gestalten in Ruhe und im stationären Zustand: Eine naturphilosophische Untersuchung*. Braunschweig, Germany: Friedr. Vieweg & Sohn.

Révész, G. (1907a). Über die Abhängigkeit der Farbenschwellen von der achromatischen Erregung. *Zeitschr. f. Sinnesphysiologie, 41*, 1-36.

Révész, G. (1907b). Über die vom Weiß ausgehende Schwächung der Wirksamkeit farbiger Lichtreize. *Zeitschr. f. Sinnesphysiologie, 41*, 102-118.

Révész, G. (1909). Über das kritische Grau. *Zeitschr. f. Sinnesphysiologie, 43*, 345-363.

Rubin, E. (1920). *Visuell wahrgenommene Figuren* Kopenhagen, Denmark: Gyldendalske Boghandel.

Seffers, K. (1922). Experimentelle Beiträge zur Untersuchung der Abhängigkeit der Unterschiedsschwelle für Helligkeiten von der antagonistischen Induktion. *Zeitschr. f. Sinnesphysiologie, 53*, 255-263.

Stumpf, C. (1917). *Die Attribute der Gesichtsempfindungen.* Preußische Akademie der Wissenschaften, Berlin.
